# The 100 Top-Cited Studies on Loneliness: A Bibliometric Analysis

**DOI:** 10.7759/cureus.37246

**Published:** 2023-04-07

**Authors:** Aditya Banerjee, Sarabjeet Kaur Chawla, Neena Kohli

**Affiliations:** 1 Department of Psychology, University of Allahabad, Prayagraj, IND

**Keywords:** citation analysis, top-cited, review, bibliometric analysis, loneliness

## Abstract

The present study explores characteristics of the top 100 most-cited articles on loneliness. A systematic search was carried out using the Thomson Reuters Web of Science Core Collection to collect studies on loneliness from inception to June 1, 2022. The initial search resulted in 6,250 search results, which included articles, book chapters, conference proceedings, editorials, and letters. Two authors independently screened the literature and extracted the data. The study supervisor removed any discrepancies. Top 100 papers (articles and reviews) on loneliness published in English were extracted. Data analysis and visualization were performed on Excel, Web of Science (WoS) Data Analyzer, and VOSviewer 1.6.16. The total number of citations of the 100 top-cited articles was 42,044, ranging from 203 to 2,201 per article. All of the studies were published from 1989 to 2021, and the years with the highest number of top-cited articles published were 2003 and 2008. Most publications were from the following journals: Computers in Human Behavior, Developmental Psychology, Psychological Science, Psychology, and Aging (n=4 each). The most cited article was titled “UCLA Loneliness Scale (Version 3): Reliability, validity, and factor structure” by Russell, DW, in the Journal of Personality Assessment. The most productive institute was the University of Chicago. The two most productive authors were Cacioppo, JT, and Hawkley, LC. Of the 100 top-cited publications, 87 were original articles and 13 were reviews. The top three WoS categories were psychology multidisciplinary, gerontology, and psychiatry. In total, 37 author keywords were elicited and further clubbed into eight distinct clusters. The study provides new insight into loneliness research, which may help doctors, researchers, and stakeholders achieve a more comprehensive understanding of trends and influential contributions to the field and highlight under-researched areas, which could be the basis for future investigation.

## Introduction and background

Although many definitions exist [1], loneliness is generally defined as the "unpleasant experience that occurs when a person's network of social relations is deficient in some important way, either quantitatively or qualitatively" [2]. Loneliness is linked to early mortality risk [3], depressive symptoms [4,5], and sleep disturbance [6], which leads to increased use of health services [7], and is found in both men and women [8] across ages [9-14]. The issues of loneliness have increased during COVID-19 [15-17]. Even prior to COVID-19, loneliness was considered one of the emerging public health concerns, and increasing attention was being paid to loneliness due to an improved understanding of the impacts it has on individuals and communities, as highlighted by the appointment of the Ministry of Loneliness in the UK and Japan [18]. With the increased interest in loneliness research, many are published yearly. Thus, a bibliometric analysis helps summarize the existing research and elicit key insights into loneliness research.

Bibliometric analysis is an emerging field that evaluates a series of academic publications such as journal articles, chapters, and other scientific publications to provide evidence and insights into a field, subject, or discipline [19]. It enables external quality evaluation of studies published within a defined timeline. It also enables progress mapping, identifying trends, and gap finding for future studies [20]. Bibliometric analysis assesses authors, institutions, and countries of research articles by mapping the structure and dynamics of discipline using a specific source [21].

The bibliometric analysis focuses on data from academic databases such as the Web of Science Core Collection (WoSCC), Scopus, and Dimension. One of the frequent types of bibliometric analysis, citation analysis, informs that the frequency of an article mentioned reflects its relative importance within that area. Recognizing the highly referenced papers and emerging papers in a field may help researchers point them in new directions. Since predatory journals are difficult to track, citation analysis helps find premier authentic journals with authors with the most impact in the field. Several bibliometric publications have recently focused on identifying the top 100 studies in a field. The focus has been on fields that publish too many papers. Therefore, keeping track of the latest development and pioneer works within a field is essential. Bibliometric analysis is a helpful tool to get an overview of major public health concerns such as cancer [22], diabetes [23], rheumatoid arthritis [24], global malnutrition [25], and ageing [26], along with new emerging fields such as neuropsychology [20], neuropathic pain [27], and studies focusing on COVID-19 [28-30]. However, to our knowledge, there has been no bibliometric analysis of loneliness research. This review provides an evidence-based reference for the most cited loneliness publications. As a result, the objective of this paper is to identify and assess the bibliometric characteristics of the top 100 loneliness-related articles.

## Review

Search strategy

**Figure 1:**
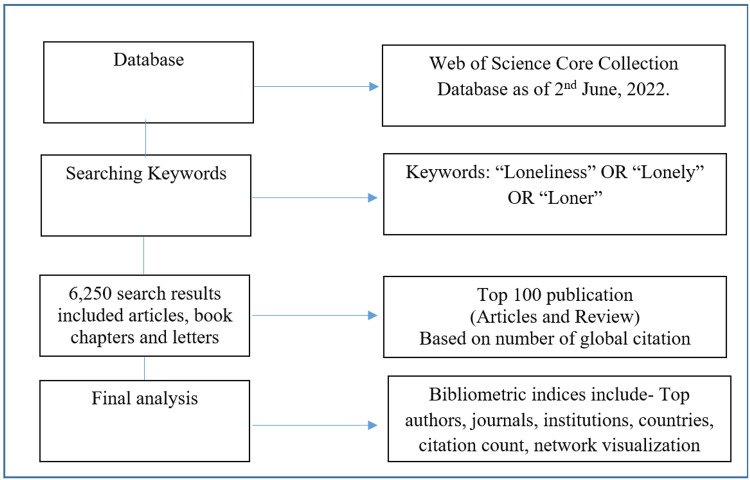
Search strategy and extraction of data from Web of Science

We searched the WoSCC database to collect articles on loneliness from inception to June 1, 2022 (Figure 1). We used the following keyword in the title search without restrictions: "Loneliness" OR "Lonely" OR "Loner." The initial search yielded 6,250 results, which included articles, book chapters, letters, and conference proceedings. Top 100 publications (articles and review papers) were extracted. The top 100 articles were ordered in descending order according to the total number of citations. The more recent article was rated higher if two articles had the same number of citations. Articles included that were published in indexed journals in English. editorials, letters, proceedings, meeting reports, and books were excluded. Two authors independently screened the title, abstract, and full text of each article on the list until the top 100 studies were identified. Disagreements were worked out with the study supervisor. The top 100 most-cited papers on loneliness were then subjected to bibliometric analysis.

Data extraction

Two authors extracted the following data independently: information regarding study title, year of publications, authors, type of publications, journal name, frequently used keywords, institutions, and countries. The first was recorded in the case of multiple corresponding authors, affiliations, or categories.

Data analysis

The data were exported to SPSS (IBM Corp., Armonk, NY) for data analysis. A map based on bibliometric data was made on VOSviewer 1.6.16 software for Windows for network visualization mapping. The retrieved data were plotted for co-authorship countries and co-occurrence of author keywords. In this study, no human or animal objects were directly involved; therefore, no ethical considerations were needed.

 Results

Table 1 lists the 100 top-cited publications in loneliness according to the total number of citations, ranked in descending order. The top 100 articles were cited 42,044 times, with an average of 420.44 citations, ranging from 203 to 2,201. The most frequently cited article, which received 2,201 citations, was published in the Journal of Personality Assessment in 1996.

**Table 1:** Top 100 most cited articles in loneliness research

Rank	Title	Authors	Journal	Year	Times Cited	Document Type
1	UCLA Loneliness Scale (Version 3): Reliability, validity, and factor structure [31]	Russell	Journal of Personality Assessment	1996	2201	Article
2	Loneliness and Social Isolation as Risk Factors for Mortality: A Meta-Analytic Review [3]	Holt-Lunstad et al.	Perspectives on Psychological Science	2015	1906	Article
3	A short scale for measuring loneliness in large surveys - Results from two population-based studies [32]	Hughes et al.	Research On Aging	2004	1498	Article
4	Loneliness Matters: A Theoretical and Empirical Review of Consequences and Mechanisms [33]	Hawkley and Cacioppo	Annals of Behavioral Medicine	2010	1469	Article
5	Friendship And Friendship Quality In Middle Childhood - Links With Peer Group Acceptance And Feelings Of Loneliness And Social Dissatisfaction [34]	Parker and Asher	Developmental Psychology	1993	1183	Article
6	Loneliness as a specific risk factor for depressive symptoms: Cross-sectional and longitudinal analyses [4]	Cacioppo et al.	Psychology and Aging	2006	1080	Article
7	Social isolation, loneliness, and all-cause mortality in older men and women [13]	Steptoe et al.	Proceedings of The National Academy of Sciences of The United States of America	2013	987	Article
8	The clinical significance of loneliness: A literature review [11]	Heinrich and Gullone	Clinical Psychology Review	2006	827	Review
9	Perceived Social Isolation Makes Me Sad: 5-Year Cross-Lagged Analyses of Loneliness and Depressive Symptomatology in the Chicago Health, Aging, and Social Relations Study [35]	Cacioppo et al.	Psychology and Aging	2010	807	Article
10	A Meta-Analysis of Interventions to Reduce Loneliness [36]	Masi et al.	Personality and Social Psychology Review	2011	734	Article
11	Loneliness and Risk of Alzheimer Disease [37]	Wilson et al.	Archives of General Psychiatry	2007	685	Article
12	An overview of systematic reviews on the public health consequences of social isolation and loneliness [38]	Leigh-Hunt et al.	Public Health	2017	622	Review
13	Who uses Facebook? An investigation into the relationship between the Big Five, shyness, narcissism, loneliness, and Facebook usage [39]	Ryan and Xenos	Computers in Human Behavior	2011	617	Article
14	Loneliness and Health: Potential Mechanisms [40]	Cacioppo et al.	Psychosomatic Medicine	2002	604	Article
15	Loneliness and social isolation as risk factors for coronary heart disease and stroke: systematic review and meta-analysis of longitudinal observational studies [41]	Valtorta et al.	Heart	2016	601	Article
16	Loneliness, health, and mortality in old age: A national longitudinal study [42]	Luo et al.	Social Science & Medicine	2012	599	Article
17	Rapid Systematic Review: The Impact of Social Isolation and Loneliness on the Mental Health of Children and Adolescents in the Context of COVID-19 [15]	Loades et al.	Journal of The American Academy of Child and Adolescent Psychiatry	2020	580	Review
18	Influences on Loneliness in Older Adults: A Meta-Analysis [43]	Pinquart and Sorensen	Basic and Applied Social Psychology	2001	570	Review
19	Loneliness in Older Persons A Predictor of Functional Decline and Death [44]	Perissinotto et al.	Archives of Internal Medicine	2012	563	Article
20	Preventing social isolation and loneliness among older people: a systematic review of health promotion interventions [45]	Cattan et al.	Ageing & Society	2005	534	Review
21	A 6-Item Scale for Overall, Emotional, and Social Loneliness: Confirmatory Tests on Survey Data [46]	Gierveld and Van Tilburg	Research on Aging	2006	518	Article
22	Loneliness and Friendship in High-Functioning Children with Autism. [47]	Bauminger and Kasari	Child Development	2000	498	Article
23	Relations Among Loneliness, Social Anxiety, and Problematic Internet Use [48]	Caplan	Cyberpsychology & Behavior	2007	474	Article
24	Loneliness, Social Isolation, and Behavioral and Biological Health Indicators in Older Adults [49]	Shankar et al.	Health Psychology	2011	451	Article
25	Social isolation, loneliness and health in old age: a scoping review [50]	Courtin and Knapp	Health & Social Care in The Community	2017	442	Review
26	Loneliness: Clinical Import and Interventions [51]	Cacioppo et al.	Perspectives on Psychological Science	2015	409	Article
27	Loneliness within a nomological net: An evolutionary perspective [52]	Cacioppo et al.	Journal of Research in Personality	2006	405	Article
28	The Roles Of Social Withdrawal, Peer Rejection, And Victimization By Peers In Predicting Loneliness And Depressed Mood In Childhood [53]	Boivin et al.	Development and Psychopathology	1995	396	Article
29	Loneliness as the Cause and the Effect of Problematic Internet Use: The Relationship between Internet Use and Psychological Well-Being [54]	Kim et al.	Cyberpsychology & Behavior	2009	373	Article
30	The Prevalence of Loneliness Among Adults: A Case Study of the United Kingdom [14]	Victor and Yang	Journal of Psychology	2012	369	Article
31	The relation of social isolation, loneliness, and social support to disease outcomes among the elderly [55]	Tomaka et al.	Journal of Aging And Health	2006	369	Article
32	Alone in the Crowd: The Structure and Spread of Loneliness in a Large Social Network [56]	Cacioppo et al.	Journal Of Personality And Social Psychology	2009	368	Article
33	Loneliness And Peer Relations in Young-Children [9]	Cassidy and Asher	Child Development	1992	366	Article
34	Loneliness and the health of older people [57]	Luanaigh and Lawlor	International Journal of Geriatric Psychiatry	2008	365	Review
35	Loneliness and social uses of the internet [58]	Morahan-Martin and Schumacher	Computers in Human Behavior	2003	355	Article
36	In Defense of the internet: The relationship between Internet communication and depression, loneliness, self-esteem, and perceived social support [59]	Shaw and Gant	Cyberpsychology & Behavior	2002	352	Article
37	Loneliness in the general population: prevalence, determinants and relations to mental health [60]	Beutel et al.	BMC Psychiatry	2017	351	Article
38	Responses To Social Exclusion - Social Anxiety, Jealousy, Loneliness, Depression, And Low Self-Esteem [61]	Leary	Journal Of Social And Clinical Psychology	1990	350	Article
39	The Trajectory of Loneliness in Response to COVID-19 [16]	Luchetti et al.	American Psychologist	2020	335	Article
40	Cold and Lonely Does Social Exclusion Literally Feel Cold? [62]	Zhong and Leonardelli	Psychological Science	2008	332	Article
41	Predictors and subjective causes of loneliness in an aged population [63]	Savikko et al.	Archives of Gerontology and Geriatrics	2005	331	Article
42	Loneliness and neuroendocrine, cardiovascular, and inflammatory stress responses in middle-aged men and women [64]	Steptoe et al.	Psychoneuroendocrinology	2004	331	Article
43	Loneliness, social network size, and immune response to influenza vaccination in college freshmen [65]	Pressman et al.	Health Psychology	2005	327	Article
44	Creating social connection through inferential reproduction - Loneliness and perceived agency in gadgets, gods, and greyhounds [66]	Epley et al.	Psychological Science	2008	323	Article
45	Loneliness, social support networks, mood and wellbeing in community-dwelling elderly [67]	Golden et al.	International Journal Of Geriatric Psychiatry	2009	322	Article
46	Loneliness and International Students: An Australian Study [68]	Sawir et al.	Journal Of Studies In International Education	2008	320	Article
47	Loneliness Predicts Reduced Physical Activity: Cross-Sectional & Longitudinal Analyses [69]	Hawkley et al.	Health Psychology	2009	314	Article
48	The prevalence of and risk factors for, loneliness in later life: a survey of older people in Great Britain [70]	Victor et al.	Ageing & Society	2005	314	Article
49	Associations between loneliness and perceived social support and outcomes of mental health problems: a systematic review [71]	Wang et al.	BMC Psychiatry	2018	312	Review
50	Loneliness in everyday life: Cardiovascular activity, psychosocial context, and health behaviors [72]	Hawkley et al.	Journal of Personality and Social Psychology	2003	311	Article
51	Loneliness Across the Life Span [73]	Qualter et al.	Perspectives On Psychological Science	2015	310	Article
52	Social Isolation and Loneliness: Relationships With Cognitive Function During 4 Years of Follow-up in the English Longitudinal Study of Ageing [74]	Shankar et al.	Psychosomatic Medicine	2013	310	Article
53	Loneliness Predicts Increased Blood Pressure: 5-Year Cross-Lagged Analyses in Middle-Aged and Older Adults [75]	Hawkley et al.	Psychology And Aging	2010	307	Article
54	From Social Structural Factors to Perceptions of Relationship Quality and Loneliness: The Chicago Health, Aging, and Social Relations Study [76]	Hawkley et al.	Journals of Gerontology Series B-Psychological Sciences And Social Sciences	2008	301	Article
55	Social Isolation, Loneliness and Health Among Older Adults [77]	Coyle and Dugan	Journal of Aging and Health	2012	296	Article
56	Loneliness as a Public Health Issue: The Impact of Loneliness on Health Care Utilization Among Older Adults [78]	Gerst-Emerson et al.	American Journal of Public Health	2015	295	Article
57	Loneliness and Health in Older Adults: A Mini-Review and Synthesis [79]	Ong et al.	Gerontology	2016	292	Review
58	Feelings of loneliness, but not social isolation, predict dementia onset: results from the Amsterdam Study of the Elderly (AMSTEL) [80]	Holwerda et al.	Journal of Neurology Neurosurgery and Psychiatry	2014	284	Article
59	Social media and loneliness: Why an Instagram picture may be worth more than a thousand Twitter words [81]	Pittman and Reich	Computers in Human Behavior	2016	280	Article
60	Mindfulness-Based Stress Reduction training reduces loneliness and pro-inflammatory gene expression in older adults: A small randomised controlled trial [82]	Creswell et al.	Brain Behavior and Immunity	2012	280	Article
61	Peer Rejection In Middle School - Subgroup Differences In Behavior, Loneliness, And Interpersonal Concerns [83]	Parkhurst and Asher	Developmental Psychology	1992	276	Article
62	Social support deficits, loneliness and life events as risk factors for depression in old age. The Gospel Oak Project VI [84]	Prince et al.	Psychological Medicine	1997	275	Article
63	Peer interaction and loneliness in high-functioning children with autism [85]	Bauminger	Journal of Autism And Developmental Disorders	2003	271	Article
64	Impact of Internet Use on Loneliness and Contact with Others Among Older Adults: Cross-Sectional Analysis [86]	Cotten et al.	Journal Of Medical Internet Research	2013	270	Article
65	The De Jong Gierveld short scales for emotional and social loneliness: tested on data from 7 countries in the U.N. generations and gender surveys [87]	De Jong Gierveld and Van Tilburg	European Journal Of Ageing	2010	269	Article
66	Loneliness is a unique predictor of age-related differences in systolic blood pressure [88]	Hawkley et al.	Psychology And Aging	2006	266	Article
67	Anxiety, Social Deficits, and Loneliness in Youth with Autism Spectrum Disorders [89]	White and Roberson-Nay	Journal Of Autism And Developmental Disorders	2009	261	Article
68	Lonely hearts: Psychological perspectives on loneliness [90]	Ernst and Cacioppo	Applied & Preventive Psychology	1999	259	Review
69	Interventions to reduce social isolation and loneliness among older people: an integrative review [91]	Gardiner et al.	Health & Social Care In The Community	2018	254	Review
70	Functional Analyses of LONELY GUY Cytokinin-Activating Enzymes Reveal the Importance of the Direct Activation Pathway in Arabidopsis [92]	Kuroha et al.	The Plant Cell	2009	249	Article
71	Loneliness and depression in independent living retirement communities: risk and resilience factors [93]	Adams et al.	Aging & Mental Health	2004	249	Article
72	Do Lonely Days Invade the Nights? Potential Social Modulation of Sleep Efficiency [94]	Cacioppo et al.	Psychological Science	2002	248	Article
73	Changes in Older Adult Loneliness: Results From a Seven-Year Longitudinal Study [95]	Dykstra et al.	Research On Aging	2005	242	Article
74	Animal-Assisted Therapy and Loneliness in Nursing Homes: Use of Robotic versus Living Dogs [96]	Banks et al.	Journal Of The American Medical Directors Association	2008	241	Article
75	Loneliness and Internet use [97]	Amichai-Hamburger and Ben-Artzi	Computers In Human Behavior	2003	241	Article
76	The Lonely Superpower [98]	Huntington	Foreign Affairs	1999	236	Article
77	Social Isolation and Loneliness in Old Age: Review and Model Refinement [99]	Wenger et al.	Ageing And Society	1996	235	Review
78	Sense of community referred to the whole town: Its relations with neighboring, loneliness, life satisfaction, and area of residence [100]	Prezza et al.	Journal Of Community Psychology	2001	234	Article
79	Counteracting Loneliness: On the Restorative Function of Nostalgia [101]	Zhou et al.	Psychological Science	2008	232	Article
80	Age Differences in Loneliness From Late Adolescence to Oldest Old Age [102]	Luhmann and Hawkley	Developmental Psychology	2016	230	Article
81	Loneliness and HIV-related stigma explain depression among older HIV-positive adults [103]	Grov et al.	Aids Care-Psychological And Socio-Medical Aspects Of Aids/Hiv	2010	230	Article
82	Correlates and predictors of loneliness in older-adults: a review of quantitative results informed by qualitative insights [104]	Cohen-Mansfield et al.	International Psychogeriatrics	2016	226	Review
83	Loneliness and peer relations in childhood [105]	Asher and Paquette	Current Directions In Psychological Science	2003	226	Article
84	Loneliness in the U.K. during the COVID-19 pandemic: Cross-sectional results from the COVID-19 Psychological Wellbeing Study [106]	Groarke et al.	PLOS ONE	2020	224	Article
85	Loneliness, health and depression in older males [107]	Alpass and Neville	Aging & Mental Health	2003	224	Article
86	Age and loneliness in 25 European nations [108]	Yang and Victor	Ageing & Society	2011	223	Article
87	Popularity, Friendship Quantity, and Friendship Quality: Interactive Influences on Children’s Loneliness and Depression [109]	Nangle et al.	Journal Of Clinical Child And Adolescent Psychology	2003	222	Article
88	Need for belonging, relationship satisfaction, loneliness, and life satisfaction [110]	Mellor et al.	Personality And Individual Differences	2008	221	Article
89	We Are Staying at Home. Association of Self-perceptions of Aging, Personal and Family Resources, and Loneliness With Psychological Distress During the Lock-Down Period of COVID-19 [17]	Losada-Baltar et al.	Journals Of Gerontology Series B-Psychological Sciences And Social Sciences	2021	220	Article
90	Intimacy, Loneliness and Sexual Offenders [111]	Marshall	Behaviour Research And Therapy	1989	220	Article
91	Linking Loneliness, Shyness, Smartphone Addiction Symptoms, and Patterns of Smartphone Use to Social Capital [112]	Bian and Leung	Social Science Computer Review	2015	217	Article
92	The Anatomy of Loneliness [113]	Cacioppo et al.	Current Directions In Psychological Science	2003	209	Article
93	Social Contacts and Their Relationship to Loneliness among Aged People - A Population-Based Study [114]	Routasalo et al.	Gerontology	2006	207	Article
94	Toward a Neurology of Loneliness [115]	Cacioppo et al.	Psychological Bulletin	2014	206	Article
95	Does Posting Facebook Status Updates Increase or Decrease Loneliness? An Online Social Networking Experiment [116]	Deters and Mehl	Social Psychological And Personality Science	2013	206	Article
96	Loneliness in Relation to Suicide Ideation and Parasuicide: A Population-Wide Study [117]	Stravynski and Boyer	Suicide And Life-Threatening Behavior	2001	206	Article
97	Chronicity and Instability of Children’s Peer Victimization Experiences as Predictors of Loneliness and Social Satisfaction Trajectories [118]	Kochenderfer-Ladd and Wardrop	Child Development	2001	204	Article
98	Children’s perceptions of their peer experiences: Attributions, loneliness, social anxiety, and social avoidance [119]	Crick and Ladd	Developmental Psychology	1993	204	Article
99	Prevalence and predictors of general psychiatric disorders and loneliness during COVID-19 in the United Kingdom [120]	Li and Wang	Psychiatry Research	2020	203	Article
100	The effects of sense of belonging, social support, conflict, and loneliness on depression [121]	Hagerty and Williams	Nursing Research	1999	203	Article

Year of publication

**Figure 2:**
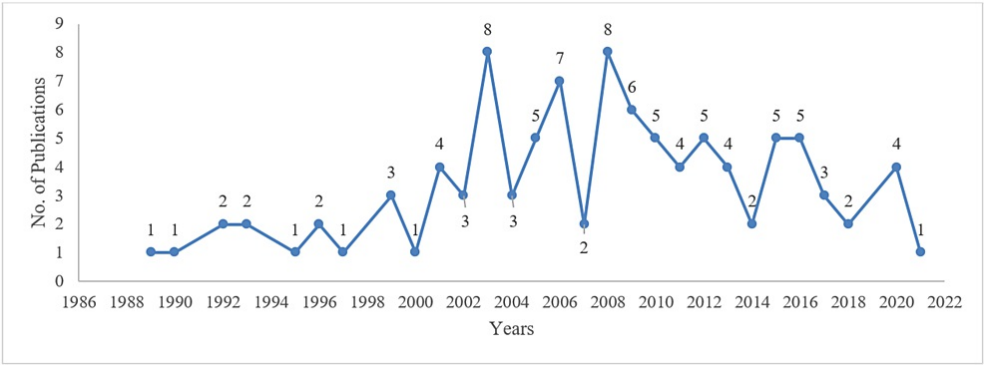
Publication by year of top 100 loneliness articles

**Figure 3:**
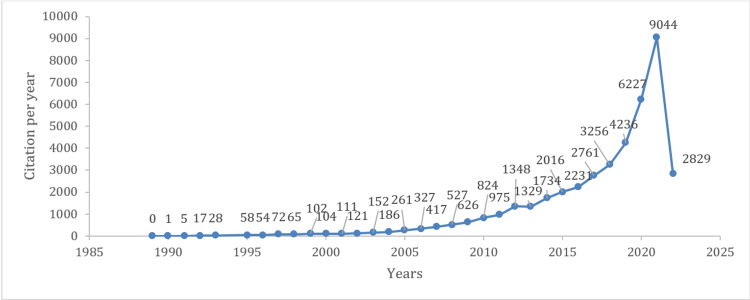
Citation per year of top 100 loneliness articles

Figure 2 shows that all of the top 100 studies were published from 1989 to 2021, and the year with the highest number of top-cited articles published was 2003 and 2008, with eight publications each, followed by seven publications in 2006. Moreover, 2008 to 2012 was the most significant five-year period, which produced 28 publications. Regarding citation per year of the top 100 studies, Figure 3 shows an upward trend in loneliness citation, with the year 2021 having the highest citation of 9,044 and an average citation of 1,274 per year.

Journals

The top 100 articles were published in 65 journals. The journals that had at least two publications are listed in Table 2. Psychology and Aging, Developmental Psychology, Computers in Human Behavior, and Psychological Science had the highest publication (n=4) each. The top 10 journals published 34 articles cumulatively, and 44 journals had a single publication in the list of the top 100. The impact factor of journals ranged from 2.16 (Journal of Aging and Health) to 8.19 (Perspectives on Psychological Science). We did not find a relationship between the number of published studies and the journal's impact factor (p > 0.05). Also, we did not find a relationship between the average number of citations for each journal and the journal's impact factor (p > 0.05).

**Table 2:** Journals that published at least two of the 100 top-cited papers. Latest journal impact factors were updated from the journal homepage.

Journal	Number of Studies	Total Citation	Average Citation	Impact Factor (Year)
Psychology and Aging	4	2,463	615.7	4.23 (2020)
Developmental Psychology	4	1,893	473.2	3.84 (2020)
Computers in Human Behavior	4	1,494	373.5	6.83 (2020)
Psychological Science	4	1,135	283.7	4.90 (2018)
Perspectives on Psychological Science	3	2,628	876	8.19 (2018)
Research on Aging	3	2,262	754	2.38 (N.A.)
Cyberpsychology & Behavior	3	1,199	399.6	4.15 (2020)
Health Psychology	3	1,093	364.3	3.53 (2018)
Ageing & Society	3	1,071	357	3.71 (2020)
Child Development	3	1,068	356	5.02 (2018)
Psychosomatic Medicine	2	914	457	4.31 (2020)
Health Social Care in the Community	2	696	348	2.82 (2020)
International Journal of Geriatric Psychiatry	2	687	343.5	3.48 (2020)
Journal of Personality and Social Psychology	2	679	339.5	7.67 (2020)
Journal of Aging and Health	2	665	332.5	2.16 (2016)
BMC Psychiatry	2	664	332	4.14 (2021)
Journal of Autism and Developmental Disorders	2	533	266.5	3.04 (2019)
Journals of Gerontology Series B Psychological Sciences and Social Sciences	2	521	260.5	4.07 (2022)
Gerontology	2	499	249.5	5.59 (N.A.)
Aging Mental Health	2	473	236.5	3.65 (2020)
Current Directions in Psychological Science	2	435	217.5	4.67 (2017)

Country

**Figure 4:**
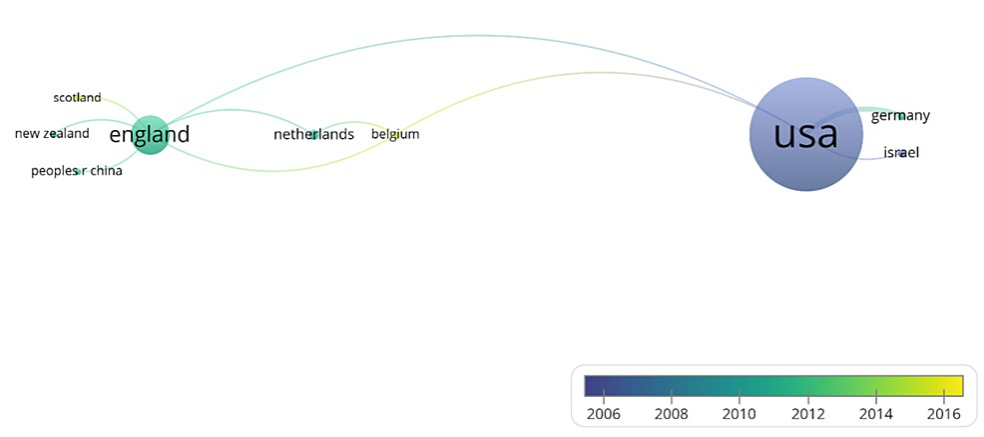
Network overlay of co-authorship map of country of top 100 publications

A total of 16 countries (excluding Belgium and Scotland) contributed to the top 100 cited publications (Table 3). The most productive countries were the USA (n=51), England (n=17), Australia, Canada, Germany, Israel, and the Netherlands (n=4). The other countries contributed to less than three publications. The largest set of connected countries consisted of nine countries that could be divided into three distinct clusters (Figure 4). The first cluster consisted of England, New Zealand, China, and Scotland; the second cluster consisted of the USA, Germany, and Israel; and the third cluster consisted of the Netherlands and Belgium. Network overlay visualizations of the co-authorship of countries are shown in Figure 3. The USA dominated the earliest publications, with recent publications from Belgium and Scotland.

**Table 3:** Countries of 100 top-cited publications. If a study had a different first and corresponding author's country, the corresponding author's country was considered in the list.

Countries	Record Count	Total Citation	Average Citation
USA	51	24,821	486.68
England	17	7,118	418.7
Australia	4	1,313	328.25
Canada	4	1,236	309
Germany	4	1,357	339.25
Israel	4	1,154	288.5
Netherlands	4	1,985	496.25
Finland	2	687	343.5
Ireland	2	538	269
China	2	449	224.5
Italy	1	235	235
Japan	1	220	220
New Zealand	1	224	224
North Ireland	1	224	224
Spain	1	249	249
Wales	1	234	234

Authors

**Figure 5:**
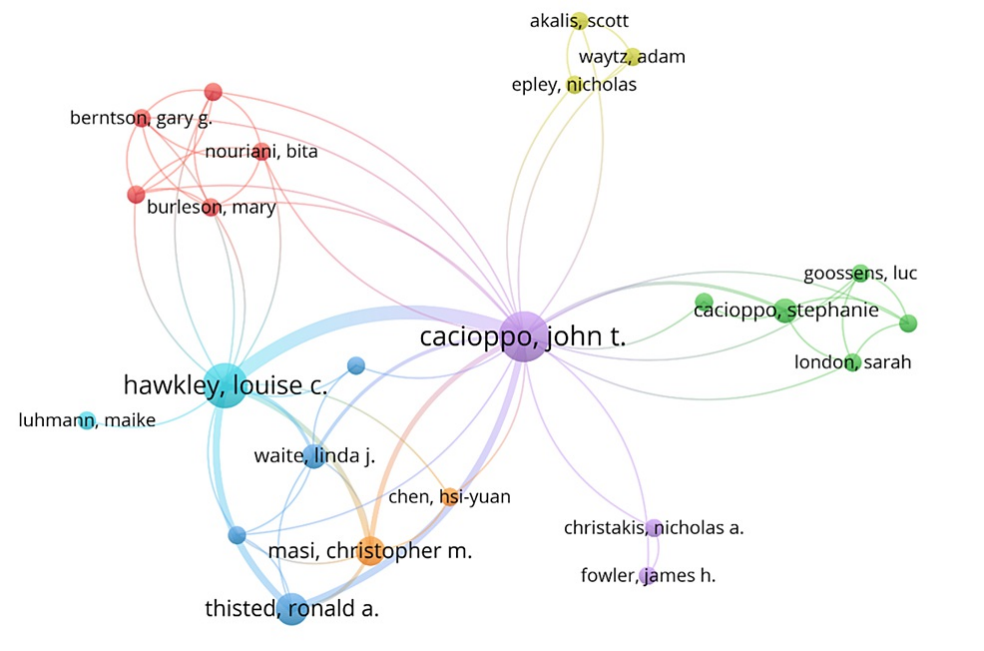
Co-authorship map of authors

Nine authors published at least two top-cited papers as first or corresponding authors listed in Table 4. Hawkley and Cacioppo published seven publications, each as either the first author or corresponding author. They also published most papers together. The co-authorship map of authors in Figure 5 shows that Cacioppo is the most influential author in loneliness publications.

**Table 4:** Authors who published at least two papers as the first or corresponding author

Author Position	Name	Number of Studies
First author	Cacioppo, JT	7
	Hawkley, LC	6
	Cacioppo, S	2
	Gierveld, JD	2
	Bauminger, N	2
	Shankar, A	2
	Steptoe, A	2
	Victor, CR	2
Corresponding author	Hawkley, LC	7
	Cacioppo, JT	6
	Bauminger, N	2
	Cacioppo, S	2
	Gierveld, JD	2
	Pitkala, KH	2
	Shankar, A	2
	Steptoe, A	2
	Victor, CR	2

Institute

A total of nine institutes published at least two or more top-cited papers (Table 5). University of Chicago, Chicago, IL, USA, published most papers (n=17), followed by the University of London, England (n=7), and the University of Illinois System, Urbana, IL, USA. The top two institutes published 24% of papers.

**Table 5:** Institutes that published at least two of the top 100 top cited studies The institute of the first author was used for data analysis

Institute	Country	Number of Studies	Total Citation
University of Chicago	USA	17	8,361
University of London	England	7	3,108
University of Illinois System	USA	4	2,029
Bar-Ilan University	Israel	3	1,010
Carnegie Mellon University	USA	2	607
Royal Netherlands Academy of Arts Sciences	Netherlands	2	760
University of Michigan System	USA	2	555
Duke University	USA	2	1,724
Vrije Universiteit Amsterdam	Netherlands	2	443

Study type and Web of Science categories

Of the top 100 cited publications, 87 were original articles and 13 were reviews (Table 6). The average citation for articles was 420.1 and that for reviews was 424.7. Eighteen Web of Science (WoS) categories had at least two top-cited papers. The top three categories were psychology multidisciplinary (n=24), gerontology (n=21), and psychiatry (n=17).

**Table 6:** Publication by WOS category of top-cited papers with at least two papers If an article had more than one WoS category, both categories were used for data analysis. WoS, Web of Science

Type of Study	Number of studies	Total Citation	Average Citation
Articles	87	36,549	420.1
Review	13	5,521	424.7
WoS categories			
Psychology multidisciplinary	24	10,177	424.04
Gerontology	21	8,873	422.52
Psychiatry	17	5,811	341.82
Psychology developmental	16	7,156	447.25
Geriatrics gerontology	11	2,979	270.81
Psychology clinical	11	5,675	515.90
Psychology	10	3,236	323.6
Psychology social	9	5,369	596.55
Public environmental occupational health	7	2,677	382.43
Psychology applied	4	1,458	364.5
Psychology experimental	4	1,494	373.5
Communication	3	1,199	399.66
Health policy services	3	895	298.33
Psychology educational	3	1,068	356
Social work	3	930	310
Multidisciplinary sciences	2	1,213	606.5
Neurosciences	2	612	306
Social sciences biomedical	2	830	415

Author keywords

**Figure 6:**
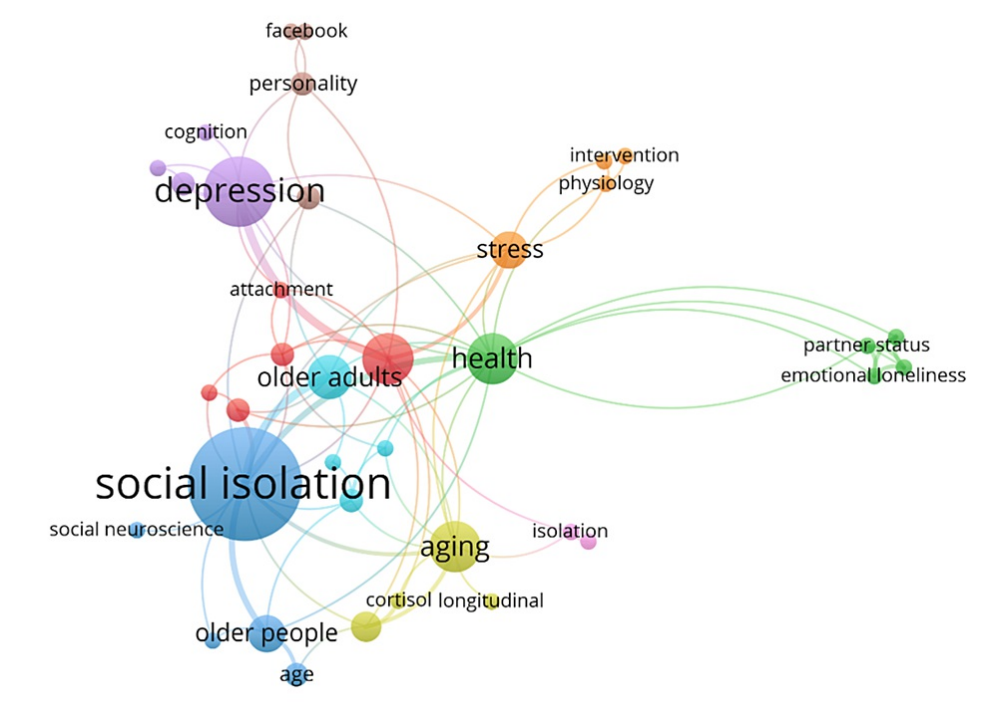
Map of co-occurrence of author keywords without the term loneliness.

The author keyword with a minimum of two occurrences and without "loneliness," which occurred in 69 titles, was used for analysis (Figure 6). A total of 37 keywords were elicited, which could be clubbed into eight distinct clusters: 1) attachment, longitudinal study, mortality, social network, and social support; 2) emotional loneliness, health, loneliness scale, partner status, and social loneliness; 3) age, older people, social isolation, social neuroscience, and systematic review; 4) ageing, blood pressure, cortisol, and longitudinal; 5) anxiety, cognition, depression, and prevalence; 6) COVID-19, loneliness risk factors, mental health, and older adults; 7) intervention, physiology, sleep, stress; 8) Facebook, Internet, personality, and shyness.

Discussion

Research in the field of loneliness has increased over time. A review of the most influential papers and authors can provide a detailed overview of research trends for researchers and practitioners. In this review, we tried identifying and assessing the bibliometric characteristics of the top 100 loneliness-related publications. To our knowledge, no citation analyses have examined publications on loneliness. Therefore, this study represents the first comprehensive analysis of published literature in the field of loneliness. WoSCC database, which provides in-depth insights into multiple study characteristics [25], initially resulted in 6,250 publications. The top 100 publications (original articles and reviews) on loneliness based on the number of citations were extracted.

The study found that the total number of citations of the 100 top-cited articles was 42,044, ranging from 203 [121] to 2,201 [31]. The average citation per document was 420.44. The most cited article was published in the Journal of Personality Assessment. The University of California, Los Angeles (UCLA) loneliness (version 3) is the most frequently used uni-dimensional measure of loneliness. The measure is a revised version of the original UCLA Loneliness Scale [122] and the Revised UCLA Loneliness Scale [123]. The scale has been used extensively for a diverse population with different norms available, showing researchers' preference to use this scale as a measurement of loneliness. The current review's finding aligns with other bibliometric studies, which found that self-report measures usually receive high citations [20]. However, the scale has been critiqued as it measures loneliness as a personality trait or a state-related entity [124]. An overview of the publication year suggests that research on loneliness is relatively new compared to other psychological issues such as depression [125,126].

All the top 100 studies were published from 1989 to 2021, and the years with the highest number of top-cited articles published were 2003 and 2008, with eight publications each followed by 2006. Also, 2008 to 2012 was the most influential five-year period, producing 28 out of the top 100 publications. There was an upward trend in loneliness citation, with the year 2021 having the highest citation of 9,044 and an average citation of 1,274 of the top 100 articles per year. One of the reasons for the growing interest in loneliness research is that COVID-19 measures such as social distancing and lockdown increased the rate of loneliness and social isolation. The author's keyword co-occurrence analysis also shows that the COVID-19-related loneliness studies have been published recently, creating significant impact and being cited significantly.

The highest number of publications was four from journals: Psychology and Aging, Developmental Psychology, Computers in Human Behavior, and Psychological Science. Top 10 journals published 34 articles cumulatively. The impact factor of journals ranged from 2.16 (Journal of Aging and Health) to 8.19 (Perspectives on Psychological Science). We did not find a relationship between the number of published studies and the journal's impact factor. Also, we did not find a relationship between average number of citations for each journal and journal's impact factor. Similar results were found in past research [20] in the study of top-cited papers in neuropsychology showing that journal impact factor is not the most important factor in journal selection.

The most productive country was the USA, followed by England. The largest set of connected countries comprised nine countries that could be divided into three distinct clusters. The first cluster consisted of England, New Zealand, China, and Scotland; the second cluster consisted of The USA, Germany, and Israel; and the third cluster consisted of the Netherlands and Belgium. Network overlay visualizations of the co-authorship of countries show the USA dominated the earliest publication, with recent publications emerging from Belgium and Scotland. The impact of developed countries such as the USA and England can be attributed to more available research resources. Moreover, most countries in top-cited papers are individualistic [127]. Past studies have shown that culture plays an important role in the experience of loneliness [128-130]. Therefore, more research and newer findings from collectivist countries can enrich our knowledge on loneliness. Hawkley and Cacioppo published seven publications, each as the first or corresponding author. Cacioppo et al. have published extensively on the relationship between loneliness and its impact on the brain and physical health, mainly focusing on an evolutionary psychology approach [131]. The most productive institute was the University of Chicago, with 17 publications, followed by the University of London, with seven publications. Out of 100 top-cited publications, 87 were original articles and 13 were reviews. The top three WoS categories were psychology multidisciplinary with 24 publications, gerontology with 21, and psychiatry with 17. Out of the top five categories, two WoS categories were of geriatrics, highlighting that loneliness has been extensively studied as a problem of the elderly. Focusing on other age groups can open new research avenues.

The co-occurrence of author keywords showed that loneliness research had been conducted in different viewpoints and fields. A total of 37 author keywords were elicited, which could be clubbed into eight distinct clusters. The first cluster comprises loneliness attachment, longitudinal study, mortality, social network, and social support. A multi-dimensional perspective of loneliness [132] dominates the second cluster with keywords such as emotional loneliness, social loneliness, health, loneliness scale, and partner status. The third cluster is dominated by the elderly and geriatric population, which consists of keywords such as age, older people, social isolation, social neuroscience, and systematic review. The fourth cluster consists of keywords from the evolutionary theory of loneliness [131], such as ageing, blood pressure, cortisol, and longitudinal. The fifth cluster comprises psychological problems such as anxiety, cognition, depression, and prevalence. The sixth cluster shows emerging topics of loneliness during COVID-19, such as loneliness risk factors, mental health, and older adults. The seventh cluster consists of intervention, physiology, sleep, and stress. The eighth cluster consists of keywords focusing on enduring traits of individuals, such as personality and shyness. This cluster also focuses on lonely individuals' online behavior and consists of keywords such as Facebook and Internet.

Limitations

The current research has some limitations. We did not collect data from other databases, such as Scopus, Medline, or Google Scholar, because we used the WoS database exclusively for our analysis. Second, we only considered research that had been published in English. Third, since the number of citations to each publication changes over time, the top 100 cited papers will also vary. Fourth, we may have missed some studies that did not contain the term "Loneliness" or "Lonely" or "Loner" in the title, as we did not consider studies where the core word was not used in the title. Fifth, citation rates are affected by many factors, many of which are beyond the scope of this study. In the future study, we will include more databases and dynamically track changes in these studies.

## Conclusions

In the present bibliometric study, we identified and analyzed the 100 top-cited publications on loneliness. With a recent surge in top-cited publications, loneliness has become a growing public issue. However, Individualized countries still dominate influential publications with leading researchers from the USA. The review provides insight into the most impactful loneliness studies and highlights critical and insufficiently investigated topics. More research from Asian countries will help practitioners and other stakeholders to enhance their understanding of the trends and influential contributions to loneliness research.
